# 20(*S*)-25-methoxyl-dammarane-3*β*, 12*β*, 20-triol, a novel natural product for prostate cancer therapy: activity *in vitro* and *in vivo* and mechanisms of action

**DOI:** 10.1038/sj.bjc.6604227

**Published:** 2008-02-05

**Authors:** W Wang, H Wang, E R Rayburn, Y Zhao, D L Hill, R Zhang

**Affiliations:** 1 Department of Pharmacology and Toxicology, Division of Clinical Pharmacology, and Comprehensive Cancer Center, University of Alabama at Birmingham, Birmingham AL 35294, USA; 2 Institute for Nutritional Sciences, Shanghai Institute of Biological Sciences, Chinese Academy of Sciences, Shanghai 200031, PR China; 3 Shenyang Pharmaceutical University, Shenyang 110016, PR China

**Keywords:** *Panax notoginseng*, 25-OCH_3_-PPD, ginsenoside, natural products, prostate cancer, chemosensitisation, radiosensitisation

## Abstract

We recently isolated 20(*S*)-25-methoxyl-dammarane-3*β*, 12*β*, 20-triol (25-OCH_3_-PPD), a natural product from *Panax notoginseng*, and demonstrated its cytotoxicity against a variety of cancer cells. Here we report the effects of this compound *in vitro* and *in vivo* on human prostate cancer cells, LNCaP (androgen-dependent) and PC3 (androgen-independent), in comparison with three structurally related ginsenosides, ginsenoside Rh2, ginsenoside Rg3, and 20(*S*)-protopanaxadiol. Of the four test compounds, 25-OCH_3_-PPD was most potent. It decreased survival, inhibited proliferation, induced apoptosis, and led to G1 cell cycle arrest in both cell lines. It also decreased the levels of proteins associated with cell proliferation (MDM2, E2F1, cyclin D1, and cdks 2 and 4) and increased or activated pro-apoptotic proteins (cleaved PARP, cleaved caspase-3, -8, and -9). In LNCaP cells, 25-OCH_3_-PPD inhibited the expression of the androgen receptor and prostate-specific antigen. Moreover, 25-OCH_3_-PPD inhibited the growth of prostate cancer xenograft tumours. Combining 25-OCH_3_-PPD with conventional chemotherapeutic agents or with radiation led to potent antitumour effects; tumour regression was almost complete following administration of 25-OCH_3_-PPD and either taxotere or gemcitabine. 25-OCH_3_-PPD also demonstrated low toxicity to noncancer cells and no observable toxicity in animals. In conclusion, our preclinical data indicate that 25-OCH_3_-PPD is a potential therapeutic agent against both androgen-dependent and androgen-independent prostate cancer.

Prostate cancer poses a major public health problem worldwide. In the United States, it is the most frequently diagnosed cancer, and the third leading cause of cancer death in men (American Cancer Society, Inc., 2007). Since androgen- and androgen receptor (AR)-mediated signalling are often essential for the initiation and progression of prostate cancer (Feldman and Feldman, 2001; Heinlein and Chang, 2004), targeting the receptor and/or its signalling pathways represents a rational strategy for treating this disease (Kelloff *et al*, 1999; Bosland *et al*, 2002). Although antiandrogens provide effective treatment for some prostate cancers, many patients develop androgen-independent tumours that are generally more aggressive, more resistant to the presently used chemotherapeutic agents, and more likely to metastasise (Tammela, 2004; Petrylak, 2005; Quinn *et al*, 2005). Thus, there is an urgent need for novel therapeutic agents to improve the outcome of antiprostate cancer therapy. Especially desirable are compounds that are structurally different from those currently used and novel agents that inhibit cell-cycle progression, induce apoptosis, and target multiple aspects of AR-dependent and AR-independent signalling.

Natural products provide a rich source for developing novel anticancer agents. We have recently been interested in evaluating the anticancer activity of ginseng and related herb medicines. Ginseng is used in many cultures, especially in China and other Asian countries, for the treatment and prevention of various diseases, including cancer (Helms, 2004). People who consume ginseng preparations are at lower risk for cancers of the oral cavity, stomach, lung, liver, pancreas, ovaries, and colon (Yun, 2003). *Panax ginseng* (Korean ginseng), *Panax quinquefolius* (American ginseng), and other related plants, including *Panax notoginseng/pseudoginseng* (*P. notoginseng*, Buck FH Chen), are frequently used for medicinal purposes. In the United States, ginseng is one of the most widely used medicinal herbs (O’Hara *et al*, 1998). Although only part of the complex mixture of compounds present in these plants, the ginsenosides (saponin triterpene glycosides), are apparently responsible for most of the pharmacological effects of ginseng and notoginseng. Some ginsenosides assert anticancer properties by decreasing DNA synthesis and angiogenesis, reducing host susceptibility to mutation, transformation, and DNA damage, and increasing immunosurveillance and apoptosis (Li, 1995; Attele *et al*, 1999; Kitts and Hu, 2000; Shibata, 2001; Xie *et al*, 2005). Ginsenosides also enhance the effects of traditional chemotherapeutic agents and protect normal host tissue from damage (Sato *et al*, 1994; Nakata *et al*, 1998; Helms, 2004; Wang *et al*, 2006; Xie *et al*, 2006).

We recently isolated a novel ginsenoside, 20(*S*)-25-methoxyl-dammarane-3*β*, 12*β*, 20-triol (25-OCH_3_-PPD), from *P. notoginseng*, and determined that it might have utility as an anticancer agent, with a better *in vitro* anticancer profile compared with other analogues (Zhao *et al*, 2007). In this study, we further evaluated this compound for its effects against prostate cancer, emphasising *in vivo* activity, molecular mechanisms, and combination therapy. In addition, to demonstrate its potency, three compounds with related structures (ginsenoside Rh2 (Rh2), ginsenoside Rg3 (Rg3), and 20(*S*)-protopanaxadiol (PPD)) were included.

## MATERIALS AND METHODS

### Test compounds

The isolation and structural characterisation of 25-OCH_3_-PPD (from *P. notoginseng*) and related compounds Rg3, Rh2, and PPD (from *P. ginseng*) were previously accomplished and described (Wang *et al*, 2007; Zhao *et al*, 2007). For isolation of 25-OCH_3_-PPD, dried leaves of *P. notoginseng* (Buck FH Chen) were extracted with 70% ethanol, and the extract was passed through a macroreticular resin column. The saponin fraction was dried, hydrolysed with 18% HCl, and extracted with ethyl acetate. The saponins were then separated by silica gel and preparative reverse-phase chromatography to yield 25-OCH_3_-PPD (Zhao *et al*, 2007), which was not found in substantial quantities in *P. ginseng*.

### Reagents

All chemicals and solvents were of the highest analytical grade available. Cell culture supplies were obtained from the Comprehensive Cancer Center Media Preparation Shared Facility (University of Alabama at Birmingham). Anti-human MDM2 (SMP14), p21 (C-19), Bcl-2 (100), Bax (N-20), E2F1 (KH95), p27 (C-19), CDK2 (M2), CDK4 (H-22), CDK6 (C-21), cyclin D1 (DCS-6), and PARP (H-250) antibodies were from Santa Cruz Biotechnology Inc. (Santa Cruz, CA, USA). The anti-human p53 (Ab-6) antibody was from EMD Chemicals Inc. (Gibbstown, NJ, USA). An anti-human antibody for prostate-specific antigen (PSA) was purchased from Dako (Glostrup, Denmark), and an anti-human antibody for the AR was purchased from BD Pharmingen (San Diego, CA, USA). The anti-human caspase-3 (9662), caspase-8 (9746), and caspase-9 (9502) antibodies were from Cell Signaling Technology Inc. (Danvers, MA, USA).

### Cell cultures

Human LNCaP (p53 wild-type, androgen-dependent) and PC3 (p53 null, androgen-independent) cells, obtained from the American Type Culture Collection (Rockville, MD, USA), were cultured as described previously (Wang *et al*, 2007). The human primary fibroblast cell lines IMR90-EEA and IMR90-E1A (transformed using the adenoviral oncogene E1A) were gifts from Dr S Lee (Harvard University, Boston, MA, USA). IMR90-EEA and IMR90-E1A cells were cultured in DMEM medium. All culture media contained 10% fetal bovine serum and 1% penicillin–streptomycin.

### Evaluations of cell survival, proliferation, apoptosis, and cell cycle distribution

For these experiments, the ginsenosides were dissolved in dimethylsulphoxide and added to the culture media so that the concentration of the solvent was 0.1%. As described previously (Wang *et al*, 2007), effects of the test compounds on human cancer cell growth and survival were determined by the use of 3-(4, 5-dimethylthiazol-2-yl)-2,5-diphenyltetrazolium bromide (MTT). Their effects on cell proliferation were evaluated by bromodeoxyuridine incorporation (Oncogene, La Jolla, CA, USA), following a previously reported protocol (Wang *et al*, 1999). Cells in the early and late stages of apoptosis were detected by use of an Annexin V-FITC apoptosis detection kit from BioVision (Mountain View, CA, USA), as previously described (Wang *et al*, 1999). For determination of the effects on the cell cycle, 2 to 3 × 10^5^ cells were exposed to the test compounds (0, 1, 5, 10, 25, or 50 *μ*M) and incubated for 24 h before analysis. Cells were trypsinised, washed with phosphate-buffered saline (PBS), and fixed in 1.5 ml of 95% ethanol at 4°C overnight, followed by incubation with RNAse and staining with propidium iodide (Sigma Chemical Co., St Louis, MO, USA). The DNA content was determined by flow cytometry. The levels of selected proteins were assessed by western blots using methods described previously (Wang *et al*, 1999). In these studies, cells were exposed to various concentrations of the four compounds for 24 h. The proteins of interest were detected by enhanced chemiluminescence reagents from Perkin Elmer LAS Inc. (Boston, MA, USA). Resveratrol, a control natural product in AR and PSA experiments, was obtained from Sigma.

### Determination of PSA protein

An assay kit (no. CM-401) from United Biotech Inc. (Mountain View, CA, USA) was used to measure secreted and cellular PSA following a method previously reported (Cho *et al*, 2004). Before exposure began, LNCaP cells, which produce PSA in response to androgen stimulation (Langeler *et al*, 1993), were washed three times with PBS to reduce background PSA. A fresh medium containing a test compound was added, and after 72 h, samples of media were collected and centrifuged to remove detached cells. In addition, cell lysates were prepared in PBS by freezing and thawing collected cells for three cycles followed by sonication for 15 s. The supernatant obtained after centrifugation (16 000 ***g***, 20 min, 4°C) was collected and stored at −20°C. For normalisation of results, protein contents of the lysates were determined by the Lowry method.

### Reverse transcriptase–polymerase chain reaction for mRNA of PSA, AR, neuron-specific enolase, and glyceraldehyde-3-phosphate dehydrogenase

A Qiagen RNeasy kit (cat no. 74104, Valencia, CA, USA) was used to extract RNA from LNCaP cells exposed to test compounds for 24 h. Reverse transcription was performed with 3–4 *μ*g total RNA and oligo(dT) primers using SuperScript™ II RT (cat no. 18064-014, GIBCO-BRL, Foster City, CA, USA). PCR was accomplished by use of the Qiagen HotStarTaq Master Mix Kit (cat no. 203445) under optimised conditions for detecting differences in transcript abundance. Oligonucleotide primers were synthesised by Sigma-Genosys (The Woodlands, TX, USA) as follows: (a) PSA (710 bp), 22 cycles; forward, 5′-GATGACTCCAGCCACGACCT-3′; reverse, 5′-CACAGACACCCCATCCTATC-3′; annealing temperature, 57°C; (b) AR (590 bp), 32 cycles; forward, 5′-ATGGAAGTGCAGTTAGGG-3′; reverse, 5′-CAGGATGTCTTTAAGGTCAGC-3′; annealing temperature, 57°C; (c) neuron-specific enolase (NSE) (662 bp) (GenBank X51956), 40 cycles; forward, 5′-GTTCTGAACGTCTGGCTAAATAC-3′; reverse, 5′-CATTGAGTTATGGGGAAATGA-3′; annealing temperature, 60°C; and (d) housekeeping gene (glyceraldehyde-3-phosphate dehydrogenase, 230 bp), 25 cycles; forward, 5′-TCAAGAAGGTGGTGAAGCAG-3′; reverse, 5′-CTTACTCCTTGGAGGCCATG-3′; annealing temperature, 57°C (Jiang *et al*, 2006).

### *In vivo* prostate cancer (PC3) xenograft model, chemotherapy, and radiation therapy

The PC3 tumour model was established as described previously (Wang *et al*, 1999). Briefly, 4- to 6-week-old male athymic nude mice (nu/nu) were obtained from the Frederick Cancer Research and Development Center (Frederick, MD, USA). Cultured PC3 cells were washed with and resuspended in serum-free medium. Portions of the suspension (5 × 10^6^ cells in 0.2 ml) were injected into the left inguinal area of the mice, which were monitored by measuring tumour growth and body weight, and by general clinical observation. Tumour-bearing mice were randomly divided into multiple treatment and control groups (*n*=5 per group). 25-OCH_3_-PPD was dissolved in PEG400/ethanol/saline (57.1% : 14.3% : 28.6%, v/v/v), and administered by intraperitoneal injection at doses of 5, 10, and 20 mg kg^−1^, 3 days per week for 4 weeks, or 5 and 10 mg kg^−1^, 5 days per week for 4 weeks. The control groups received the vehicle only. Gemcitabine (160 mg kg^−1^) and taxotere (15 mg kg^−1^) were administered by intraperitoneal injection, once a week for 2 weeks. Mice receiving radiation were first anaesthetised with a 70–100 *μ*l mixture of ketamine (20 mg ml^−1^) and xylazine (20 mg ml^−1^) at a 6.7 : 1 ratio and then placed under a specially designed lead shield so that only the tumours were exposed to the radiation beam. *γ*-Irradiation was administered by a ^60^Co Picker unit (JL Shepard Co., Glendale, CA, USA) at a rate of 0.66 Gy min^−1^. Animals received 3 Gy of radiation once a week for 3 weeks. When used in combination with gemcitabine or taxotere, 25-OCH_3_-PPD was administered at a dose of 10 mg kg^−1^ (5 days per week). Animals were monitored for activity, physical condition, body weight, and tumour growth. Tumour size was determined by caliper measurements of two perpendicular diameters of the implant every other day. Tumour weight (in grams) was estimated by use of the formula 1/2*a* × *b*^2^, where *a* is the long diameter and *b* is the short diameter (in cm). All animal protocols were approved by the Institutional Animal Care and Use Committee of UAB.

## RESULTS

### Ginsenosides decrease cancer cell growth *in vitro*

**Figure 1:**
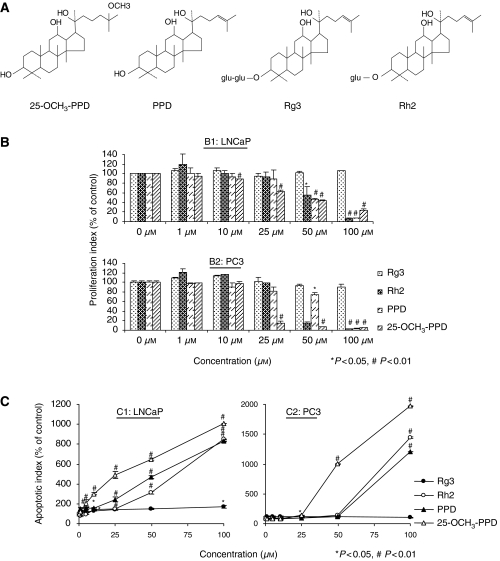
Chemical structures and anticancer activities *in vitro*. (**A**) Structures of 25-OCH_3_-PPD, PPD, Rg3, and Rh2. (**B**) Antiproliferative effects of the compounds on prostate cancer cells. LNCaP (B1) and PC3 (B2) cells were exposed to various concentrations of the four compounds for 24 h followed by assessment of cell proliferation by BrdUrd. All assays were performed in triplicate. (**C**) Induction of apoptosis in LNCaP (C1) and PC3 (C2) cells. These cells were exposed to various concentrations of ginsenosides for 48 h followed by assessment of apoptosis. All assays were performed in triplicate.

25-OCH_3_-PPD (from *P. notoginseng*) and the related *P. ginseng* compounds, PPD, Rh2, and Rg3 (Figure 1A), were evaluated for their effects on prostate cancer cell growth *in vitro* by use of the MTT assay. Following incubation with various concentrations of the compounds, amounts reducing growth by 20, 50, and 80% (IC_20_, IC_50_, and IC_80_) were calculated (Table 1). For both LNCaP (p53 wild-type, androgen-dependent) and PC3 (p53 null, androgen-independent) cells, 25-OCH_3_-PPD had the lowest IC_50_ values (in the low *μ*M range) compared with other ginsenosides tested (*P*<0.01). 20(*S*)-protopanaxadiol and Rh2 also demonstrated inhibitory effects, with values in the mid-*μ*M range. Ginsenoside Rg3 did not significantly decrease cell viability even in the high-*μ*M range. It is worth noting that Rh2 and PPD decreased the viability of two noncancer fibroblast cell lines to a comparable extent as observed for the prostate cancer cells, while 25-OCH_3_-PPD had a lesser effect on these cells (Table 1).

### Ginsenosides decrease cell proliferation

For both LNCaP and PC3 cells, an antiproliferative effect for 25-OCH_3_-PPD was evident (Figure 1B, *P*<0.001). PC3 cells were generally more sensitive to the ginsenosides than LNCaP cells. At 25 *μ*M, 25-OCH_3_-PPD inhibited LNCaP proliferation by 40% and PC3 cell proliferation by 85% (*P*<0.01). Although 25-OCH_3_-PPD was consistently most effective, Rh2 and PPD also decreased the proliferation of both cell lines (*P*<0.05); the effects of Rg3 were minimal.

### Ginsenosides induce apoptosis

In a dose-dependent manner, 25-OCH_3_-PPD induced apoptosis in both LNCaP and PC3 cells (Figure 1C). 25-OCH_3_-PPD was the most effective ginsenoside, demonstrating a more than six-fold increase in LNCaP cells and a 10-fold increase in apoptosis in PC3 cells at 50 *μ*M (*P*<0.01). A concentration of 10 *μ*M caused a three-fold increase in apoptosis in LNCaP cells. Ginsenoside Rh2 and PPD showed activity only at higher concentrations. In both cell lines, the effect of Rg3 was minimal.

### Ginsenosides cause cell cycle arrest

**Figure 2:**
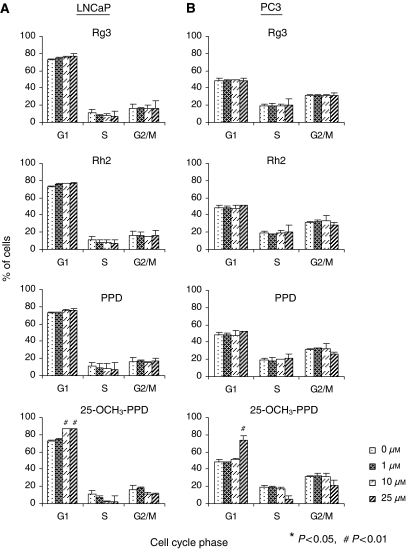
Effects of ginsenosides on cell cycle progression of prostate cancer cells. LNCaP (**A**) and PC3 (**B**) cells were exposed to various concentrations of the compounds for 24 h followed by determination of cell cycle distribution. All assays were performed in triplicate.

In both LNCaP and PC3 cells, 25-OCH_3_-PPD induced cell cycle arrest in the G1 phase in a dose-dependent manner (Figure 2, *P*<0.01). Relative to the other compounds, 25-OCH_3_-PPD (at the highest concentration, 25 *μ*M) had the most potent effects in both cell lines. 20(*S*)-protopanaxadiol and Rh2 (at the highest concentration, 50 *μ*M) altered the cell cycle, although these changes were not as great as those induced by 25-OCH_3_-PPD. As in the evaluation of cell viability, apoptosis, and proliferation, the effects of Rg3 on the cell cycle were minimal.

### Ginsenosides induce changes in expression of proteins involved in regulating apoptosis and cell cycle progression

**Figure 3:**
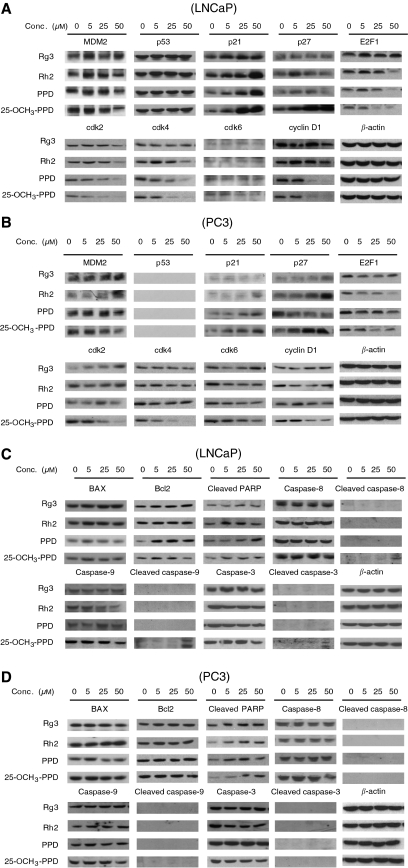
Effects of ginsenosides on expression of various cell cycle-related (**A** and **B**) and apoptosis-related (**C** and **D**) proteins in human prostate cancer cells. LNCaP (**A** and **C**) and PC3 (**B** and **D**) cells were exposed to various concentrations of the compounds for 24 h, and the target proteins were detected by immunoblotting with specific antibodies.

Possible mechanism(s) responsible for the antiproliferative, pro-apoptotic, and cell cycle regulatory effects of the four compounds were investigated by evaluating their effects on the expression of various proteins involved in these processes (Figure 3). Demonstrating how the compound affects proliferation, exposure of cells to 25-OCH_3_-PPD caused the changes in expression of several cell cycle regulatory proteins. In both LNCaP and PC3 cells, the expression levels of MDM2, E2F1, Cyclin D1, and cdks 2, 4, and 6 were decreased, whereas the expression of p21^WAF/CIP^ and p27^KIP1^ was increased (Figures 3A and B). 20(*S*)-protopanaxadiol and Rh2, which had modest effects on the growth of cultured prostate cancer cells, also altered the level of several of these proteins. Similar to cells exposed to 25-OCH_3_-PPD, the levels of p21 and cleaved PARP were increased by PPD and Rh2, and E2F1 and ckd4 were decreased. In contrast, Rg3 did not alter the expression of these proteins, with the exception of a slight increase in p21 in the LNCaP cells. The p53 protein level in LNCaP cells was not altered by any of the compounds (Figure 3A). These proteins are integral in regulating cell cycle progression and cell proliferation, and MDM2, E2F1, and cyclin D1 have been implicated in carcinogenesis (Deb, 2002; Fu *et al*, 2004; Johnson and Degregori, 2006). Downregulation of any or all of these proteins by 25-OCH_3_-PPD may lead to antiproliferative and anticancer effects.

In addition to its antiproliferative effects, we also noted that 25-OCH_3_-PPD caused an increase in apoptosis in both PC3 and LNCaP cells. We assessed the expression of several apoptosis-related proteins, including Bax, Bcl-2, cleaved PARP, and several caspases. Consistent with its effect on apoptosis, 25-OCH_3_-PPD increased the expression of cleaved PARP and cleaved caspases (Figures 3C and D). Both caspase-8 and -9 were activated, indicating that the compound induces apoptosis via both the intrinsic and extrinsic pathways. None of the three cleaved caspases were increased in PC3 cells (Figure 3D), perhaps accounting for the fact that LNCaP cells were more sensitive to the apoptosis induced by ginsenoside. This result also suggests that the effect on apoptosis may rely on p53, because LNCaP cells express wild-type p53, whereas PC3 cells are p53-null. 20(*S*)-protopanaxadiol and Rh2 affected the expression of some of the cell cycle-regulating proteins (Figures 3A and B), but to a lesser extent than 25-OCH_3_-PPD. In both cell lines, these compounds had minimal effects on apoptosis-related proteins. Ginsenoside Rg3 exposure led to a slight increase in p27, but this compound did not have an appreciable effect on any of the other proteins.

### 25-OCH_3_-PPD decreases the expression of AR and PSA

**Figure 4:**
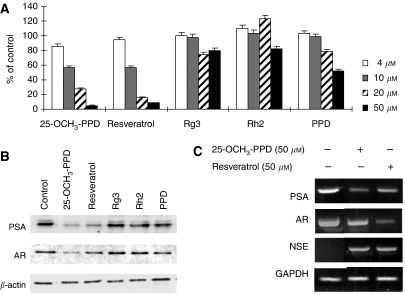
Effects on AR and PSA. (**A**) Comparison of the effects of 72-h exposure of LNCaP cells to increasing concentrations of 25-OCH_3_-PPD, Rg3, Rh2, or PPD with resveratrol on secreted PSA levels in conditioned medium. (**B**) Effects of exposure for 24 h to 50 *μ*M of 25-OCH_3_-PPD (lane 2), resveratrol (lane 3), Rg3 (lane 4), Rh2 (lane 5), and PPD (lane 6) on cellular PSA and AR protein expression, as determined by western blots. (**C**) Comparison of the effects of 25-OCH_3_-PPD with resveratrol on the steady-state level of mRNA transcripts of PSA, AR, and NSE after 24 h of exposure, as determined by reverse transcriptase–polymerase chain reaction. Neuron-specific enolase mRNA served as a molecular marker for neuroendocrine differentiation.

The effects of 25-OCH_3_-PPD, PPD, Rg3, and Rh2 on the secretion of PSA by LNCaP cells after 72 h in culture were examined. For comparison, resveratrol, a natural product known to have anti-AR activity, was included (Mitchell *et al*, 1999; Jones *et al*, 2005). 25-OCH_3_-PPD was as effective as resveratrol, decreasing the secretion of PSA in a concentration-dependent manner (Figure 4A). 20(*S*)-protopanaxadiol had a modest inhibitory effect only at 50 *μ*M; Rg3 and Rh2 did not exert any noticeable effect.

In addition, the effects of these compounds (50 *μ*M) on cellular AR and PSA protein abundance after 24 h of exposure were examined (Figure 4B). 25-OCH_3_-PPD (lane 2) substantially decreased AR and PSA levels, and PPD showed minor decreases in expression (lane 6). Neither did Rg3 (lane 4) nor Rh2 (lane 5) show any appreciable effect. The effect of 25-OCH_3_-PPD on AR protein abundance was greater than that for resveratrol. As determined by reverse transcriptase–polymerase chain reaction after 24 h of exposure of cells, 25-OCH_3_-PPD decreased both *PSA* mRNA and *AR* mRNA (Figure 4C), demonstrating that it regulates *AR* and *PSA* at the transcriptional level. In contrast, resveratrol decreased the mRNA for *PSA* but had only a minor effect on *AR* mRNA. In these cells, both 25-OCH_3_-PPD and resveratrol induced the expression of *NSE*, a marker of neuroendocrine differentiation that responds to AR inhibition (Murillo *et al*, 2001). These observations are consistent with the idea that 25-OCH_3_-PPD affects AR signalling at the AR protein level and that it has a transactivational impact on PSA mRNA transcription, leading to decreased cellular PSA synthesis and secretion into the medium.

### 25-OCH_3_-PPD inhibits the growth of human prostate xenograft tumours *in vivo*

**Figure 5:**
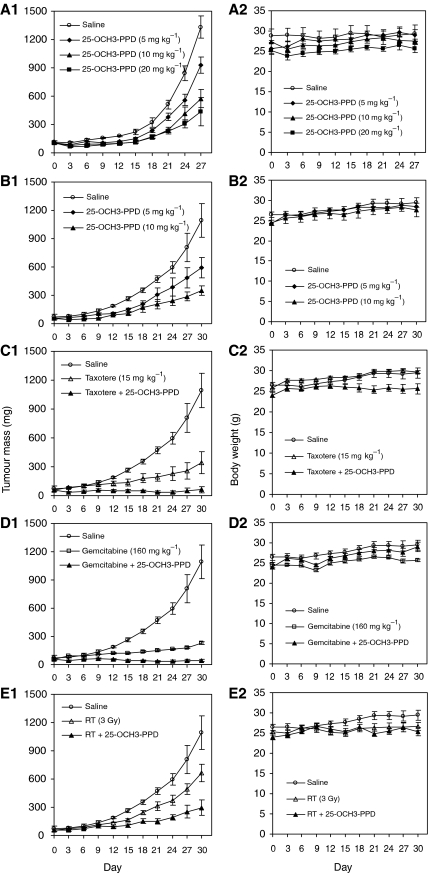
Antitumour activity and effects on body weight of 25-OCH_3_-PPD administered alone or in combination with taxotere, gemcitabine, or radiation to nude mice bearing PC3 xenograft tumours. 25-OCH_3_-PPD was given by intraperitoneal injection at doses of 5, 10, or 20 mg kg^−1^ day^−1^, 3 days per week for 4 weeks (**A1** and **A2**), or 5 or 10 mg kg^−1^ day^−1^, 5 days per week for 4 weeks (**B1** and **B2**). Taxotere (15 mg kg^−1^) (**C1** and **C2**) or gemcitabine (160 mg kg^−1^) (**D1** and **D2**) was given on days 5 and 12 by intraperitoneal injection, radiation (3 Gy) (**E1** and **E2**) was administered on days 5, 12, and 19. The dose of 25-OCH_3_-PPD was 10 mg kg^−1^, 5 days per week for studies combining ginsenoside with conventional therapy.

25-OCH_3_-PPD was evaluated in a mouse xenograft model of androgen-independent prostate cancer. The compound was first given at 5, 10, or 20 mg kg^−1^ day^−1^ 3 days per week for 4 weeks (Figure 5A1). The highest dose significantly inhibited PC3 xenograft tumour growth by 67% on day 27 (*P*<0.001). Even the lowest dose of 5 mg kg^−1^ caused significant (30%, *P*<0.05) tumour growth inhibition. In a subsequent experiment, mice received 5 or 10 mg kg^−1^ of 25-OCH_3_-PPD 5 days per week for 4 weeks. In this case, tumour growth inhibition was more substantial. Tumours in animals given the 5 mg kg^−1^ dose were only about half the size of those in control animals (*P*<0.01). At the dose of 10 mg kg^−1^, 25-OCH_3_-PPD inhibited tumour growth by 70% (Figure 5B1, *P*<0.005).

### 25-OCH_3_-PPD can be safely combined with conventional anticancer therapies

25-OCH_3_-PPD was evaluated for its effect on androgen-independent prostate cancer in combination with chemotherapeutic agents (taxotere and gemcitabine) or radiation therapy. Taxotere (15 mg kg^−1^) alone decreased tumour growth by 69%. When it was used in combination with ginsenoside, there was a significant increase in tumour growth inhibition to 94% (*P*<0.05, Figure 5C1). Similarly, although gemcitabine alone caused 80% growth inhibition, the combination of gemcitabine and 25-OCH_3_-PPD led to >96% tumour growth inhibition (Figure 5D1, *P*<0.01). Radiation alone decreased the growth of the xenograft tumours by 39%; adding ginsenoside led to more substantial inhibition (70%, *P*<0.05) (Figure 5E1).

Although mice receiving the chemotherapeutic agents or radiation experienced temporary weight loss following treatment, some of the weight was recovered afterwards. 25-OCH_3_-PPD did not cause any substantial weight loss or any observable toxic effect when administered alone and did not increase the toxicity of any of the combined therapies (Figures 5A2, B2, C2, D2 and E2).

## DISCUSSION

While cytotoxic agents are frequently used for traditional chemotherapy, there is an ongoing search for more specific agents that spare normal host tissues. Several natural products have antiangiogenic and anticancer properties (Shibata, 2001; Li *et al*, 2006; Zhang *et al*, 2006). Such compounds generally have low toxicity and can be protective against a variety of insults, such as toxic chemicals and inflammation (Kim *et al*, 1998; Smolinski and Pestka, 2003, Radad *et al*, 2004). On the basis of the assays *in vitro*, we and others have demonstrated that a structure–activity relationship exists among the ginsenosides, with the panaxadiols generally being more active than the panaxatriols, and the glycosylated compounds being less active as the number of sugar moieties increases (Shibata, 2001; Popovich and Kitts, 2002; Wang *et al*, 2007; Zhao *et al*, 2007). To our knowledge, 25-OCH_3_-PPD is one of the most active ginsenosides that have been evaluated (Zhao *et al*, 2007). More importantly, 25-OCH_3_-PPD was more cytotoxic to the prostate cancer cells than to the fibroblasts. The apparent cancer-specific effects of this compound could distinguish it from other natural products. For example, although Rh2 and PPD decreased the proliferation and viability of prostate cancer cells, they were also cytotoxic to normal (although immortalised) fibroblasts.

In confirmation of previous studies (Shibata, 2001; Kim *et al*, 2004; Cheng *et al*, 2005; Xie *et al*, 2005), Rh2 and PPD demonstrated cytotoxic, antiproliferative, pro-apoptotic, and regulatory effects on cell cycle progression. Although Rg3 is marketed as an anticancer agent, it did not exert any appreciable effect on the prostate cancer cells. It is likely that Rg3 functions as an antiangiogenic compound (Shibata, 2001; Sengupta *et al*, 2004; Yue *et al*, 2006) rather than as a cytotoxic agent. The related new compound, 25-OCH_3_-PPD, demonstrated more potent effects, and was more effective at lower doses than the other compounds. Although both prostate cancer cell lines were responsive to 25-OCH_3_-PPD (exhibiting decreases in survival and proliferation, increases in apoptosis and cell cycle arrest in G1), there were differences in their responses. These may be attributed to their differential expression of p53 or differences in sensitivity to androgen signalling.

In support of this, the differential anti-AR activities of the ginsenoside compounds evaluated in the LNCaP model appear to correlate with their growth inhibitory and pro-apoptotic efficacy. The inhibition of AR expression and signalling suggest a mechanism by which 25-OCH_3_-PPD could reduce the risk for development of prostate cancer and function in the treatment of both androgen-dependent and androgen-independent prostate cancer. Most cancers in the latter category still express wild-type AR, and ligand-independent AR signalling is essential for the survival of even androgen-refractory prostate cancer cells (Yuan *et al*, 2006). Therefore, by downregulating AR through a nontranscriptional mechanism, 25-OCH_3_-PPD could eliminate prostate cancer cells and/or make them more sensitive to chemotherapeutic drugs or radiation. Although the role of PSA in the pathobiology of prostate cancer is not fully understood (Williams *et al*, 2007), posttherapy reductions in PSA levels are associated with improved survival in patients with metastatic diseases (Fleming *et al*, 2006). Chemicals that reduce its expression, as observed for 25-OCH_3_-PPD, could be useful in treating the disease.

In addition to the effects on AR signalling, 25-OCH_3_-PPD arrested cells in the G1 phase. 25-OCH_3_-PPD and two of the other test compounds (PPD and Rh2) increased expression of p21 and cleaved PARP proteins and decreased expression of MDM2, E2F1, and cdks 2, 4, and 6. Although these results indicate that ginsenosides inhibit cell cycle progression, there were differences in the magnitude of the effects of the compounds. Individual ginsenosides may have different interactions with proteins involved in the regulation of the cell cycle. The presence of additional ginsenosides or other natural compounds may lead to undesirable or unexpected effects. These differences emphasise the necessity of strict quality control to ensure the identity, purity, and stability of the compounds.

**Figure 6:**
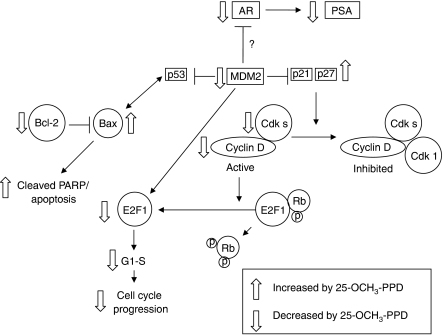
Proposed mechanisms of action. The cartoon, showing the effects of 25-OCH_3_-PPD on various proteins, demonstrates how the compound could exert its anticancer effects.

Given the effects on proliferation and apoptosis-related proteins, it is possible that downregulation of the MDM2 oncoprotein is, at least in part, responsible for the observed cytotoxic effects of 25-OCH_3_-PPD. We have previously noted that inhibition of MDM2 results in increased p21, decreased E2F1, decreased cell survival and proliferation, induction of G1 cell cycle arrest, and an increase in apoptosis, as well as *in vivo* antitumour effects (Zhang *et al*, 2003, 2004, 2005). Although further investigations are needed to identify the exact mechanism(s) of action, this study provides preliminary data about how 25-OCH_3_-PPD exerts its effects. The effects of 25-OCH_3_-PPD on various proteins and their interrelation are summarised in Figure 6.

To accomplish a preliminary evaluation of the *in vivo* therapeutic effects of 25-OCH_3_-PPD, we examined the effects of the compound alone or in combination with conventional therapies in a mouse xenograft model of androgen-independent prostate cancer. A dose of 5 mg kg^−1^ given 3 days per week led to more than 30% tumour growth inhibition. When the compound was given more frequently (5 days per week), the antitumour effect was greater, with almost 50% inhibition of tumour growth. This is noteworthy considering that ginsenosides administered intravenously typically have half-lives of less than 20 min (Qian *et al*, 2005a, 2005b), and their bioavailabilities are usually lower than 20% (Xu *et al*, 2003). Our study indicates that 25-OCH_3_-PPD is sufficiently stable to exert an anticancer effect *in vivo*, even when given only every 48 h. Nevertheless, future pharmacokinetic studies of this compound, in comparison with its analogues, will increase the understanding of its mode of action and contribute to the better design of preclinical and clinical trials.

When combined with either of the two chemotherapeutic agents, taxotere or gemcitabine, 25-OCH_3_-PPD caused almost complete tumour growth inhibition. These results are consistent with previous studies showing that ginseng compounds can be safely combined with other agents and may lead to improved antitumour activity (Wang *et al*, 2006; Xie *et al*, 2006). Similarly, the combination of radiation with 25-OCH_3_-PPD did not lead to any increase in toxicity, and there was a slight increase in tumour growth inhibition. It is possible that a longer treatment period may demonstrate a greater additive or synergistic effect. Thus, addition of the novel compound could improve the response of human tumours to radiation or to chemotherapeutic drugs that are currently used for the treatment of prostate cancer. It may also be possible to combine 25-OCH_3_-PPD with lower doses of conventional agents to achieve a strong antitumour effect, but with decreased toxic side effects. It is noteworthy that we used relatively high doses of chemotherapeutic agents in the combination study and lower doses can be used in future studies to determine if synergistic effects can be achieved between 25-OCH_3_-PPD and these agents or radiation.

Although the four ginsenosides share a common core structure, they have remarkably different effects on cancer cells. Of those tested, 25-OCH_3_-PPD demonstrated the most potent cytotoxic, antiproliferative, pro-apoptotic, and cell cycle regulatory effects. Moreover, it produced strong antitumour effects against a model of androgen-independent prostate cancer both alone and in combination with conventional cancer therapies. These results indicate that 25-OCH_3_-PPD may be an appropriate candidate for further preclinical and clinical development as an antiprostate cancer agent either alone or in combination with conventional therapies. Further studies of its efficacy, toxicity, and pharmacokinetics are necessary prior to human clinical trials. Although the compound can be purified to >99% (Zhao *et al*, 2007), a well-designed quality control procedure is needed. In addition, a synthetic method for generating 25-OCH_3_-PPD, if successful, would ensure the consistency, potency, and purity of the compound for human studies.

## Figures and Tables

**Table 1:** Growth inhibitory activity of the four ginseng compounds

**Cell line**	**Inhibitory concentration^a^**	**25-OCH_3_-PPD (*μ*M)**	**PPD (*μ*M)**	**Rg3 (*μ*M)**	**Rh2 (*μ*M)**
LNCaP	IC_20_	5.4	28.6	154.8	30.3
	IC_50_	12.0	44.8	302.1	46.7
	IC_80_	26.6	70.1	>500	72.0
					
PC3	IC_20_	1.5	12.9	176.7	15.7
	IC_50_	5.6	29.3	266.5	35.7
	IC_80_	20.6	66.6	402.0	81.4
					
IMR90-EEA	IC_20_	10.4	22.8	297.8	58.2
	IC_50_	110.1	82.6	387.1	74.4
	IC_80_	>500	298.8	>500	95.0
					
IMR90-E1A	IC_20_	6.2	1.3	52.7	28.0
	IC_50_	275.7	18.2	146.8	48.5
	IC_80_	>500	257.0	409.3	84.1

Abbreviations: PPD=20(*S*)-protopanaxadiol; Rh2=ginsenoside Rh2; Rg3=ginsenoside Rg3; 25-OCH_3_-PPD=20(*S*)-25-methoxyl-dammarane-3*β*, 12*β*, 20-triol.

a IC_20_, IC_50_, and IC_80_ are the concentrations that inhibit growth by 20, 50, and 80%, respectively, relative to the control.
